# Targeting histone lysine demethylases — Progress, challenges, and the future^☆^

**DOI:** 10.1016/j.bbagrm.2014.05.009

**Published:** 2014-12

**Authors:** Cyrille C. Thinnes, Katherine S. England, Akane Kawamura, Rasheduzzaman Chowdhury, Christopher J. Schofield, Richard J. Hopkinson

**Affiliations:** The Chemistry Research Laboratory, Mansfield Road, Oxford, OX1 3TA, UK

**Keywords:** Demethylase, Epigenetics, Methylation, Inhibition, Histone, Lysine

## Abstract

*N*-Methylation of lysine and arginine residues has emerged as a major mechanism of transcriptional regulation in eukaryotes. In humans, *N*^ε^-methyllysine residue demethylation is catalysed by two distinct subfamilies of demethylases (KDMs), the flavin-dependent KDM1 subfamily and the 2-oxoglutarate- (2OG) dependent JmjC subfamily, which both employ oxidative mechanisms. Modulation of histone methylation status is proposed to be important in epigenetic regulation and has substantial medicinal potential for the treatment of diseases including cancer and genetic disorders. This article provides an introduction to the enzymology of the KDMs and the therapeutic possibilities and challenges associated with targeting them, followed by a review of reported KDM inhibitors and their mechanisms of action from kinetic and structural perspectives. This article is part of a Special Issue entitled: Methylation: A Multifaceted Modification — looking at transcription and beyond.

## Highlights

• *N*-Methylation of histone lysines plays important roles in regulating transcription.
• Methyllysines are subject to demethylation by histone lysyl demethylases (KDMs).
• Many KDMs exhibit dysregulated expression patterns in disease.
• Inhibition of KDMs is an important therapeutic target.

## 1 Introduction

Post-oligomerisation modifications to the nucleic acid and protein components of chromatin are of central importance in eukaryotic transcriptional regulation. Established modifications to chromatin include methylation of DNA and histones, and further modifications to histones include acetylation, phosphorylation, and ubiquitination, with the *N*-terminal tail of histone H3 being subjected to a particularly complex set of modifications. These modifications, together with other factors, are proposed to substantially contribute to the regulation of gene expression in a context-dependent manner, in part by affecting the accessibility of DNA sequences for transcription [1], [2], [3]. The post-translational modifications affect chromatin structure and dynamics, the lifetimes of chromatin components, and mediate chromatin binding interactions. Aberrant modification patterns to chromatin have been associated with the onset and progression of both germline and somatic diseases, ranging from mental disorders to many cancers [4], [5], [6]. Presently, it seems quite possible that most, if not all, major human diseases (including at least some infectious diseases) will be linked to changes in post-oligomerisation modifications to chromatin. Consequently, many of the enzymes that catalyse these modifications and their removal (sometimes referred to as ‘writers’ and ‘erasers’, respectively), as well as the multiple binding domains that interact with them, are being pursued as small-molecule targets for therapeutic benefit. Small molecules targeting chromatin-modifying enzymes for cancer treatment are already in clinical use (e.g. DNA methyltransferase inhibitors (Azacitidine, Decitabine) and histone deacetylase inhibitors (Vorinostat, Romidepsin)), and others are in trials [7], [8], [9].

*N*-Methylations of both DNA and histones are chromatin modifications of central importance in (epi)genetics. Due to the strength of the C–N bond, *N*-methylation was once thought to be irreversible, at least via direct enzymatic catalysis; however, it is now clear that *N*-methylation of DNA (and RNA) is both common and can be directly reversed by the action of demethylases (note that indirect methods of repair of methylated DNA, for example base excision repair, are well-established, but beyond the scope of this article).

The available evidence is that protein *N*-methylation occurs principally, but not exclusively, on the histone lysyl and arginyl side chains of histones H3 and H4. There is also evidence, both from ‘global’ proteomic as well as focused studies, that many other proteins are *N*-methylated (and likely demethylated), including some associated with transcriptional regulation (e.g. methylation of p53 [10] and NF-κB [11], [12]). In contrast to histone lysine acetylation, which results in transcriptional activation, histone methylation can either enable context-dependent activation or repression of transcription. In general, methylation of histone H3 at Lys9 or Lys27 (H3K9 or H3K27) or histone H4 at Lys20 (H4K20) correlates with transcriptional repression, whereas methylation of H3K4, H3K36 and H3K79 correlates with enhanced transcription [13]. The outcomes of methylation depend on its site and extent, the presence of other chromatin modifications, and many other regulatory factors.

**Fig. 1:**
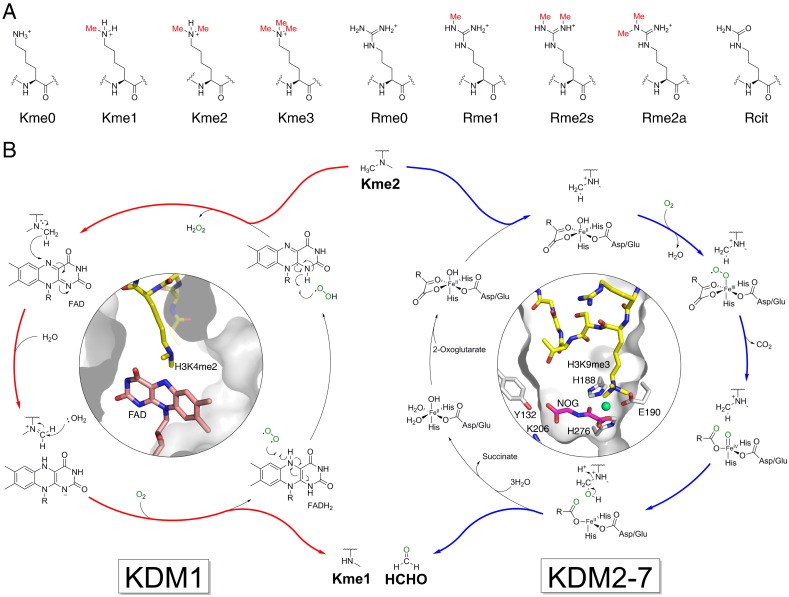
Histone lysyl demethylation is catalysed by histone demethylases (KDMs). (A) Methylation states of lysine and arginine residues in histones. Lysine residues may be mono-, di- or trimethylated on their *N^ε^*-amino groups (Kme1, Kme2 and Kme3 respectively), whereas arginine residues may be either mono- or dimethylated on their *N^ω^*-guanidino nitrogens (Rme1 and Rme2 respectively). Dimethylated arginine exists in either symmetric (Rme2s) or asymmetric (Rme2a) forms, depending upon the positions of methylation. Arginine may also be deaminated to form citrulline (Rcit), as catalysed by peptidylarginine deiminase activity. (B) Mechanisms of lysyl demethylation catalysed by the KDM1 and JmjC (KDM2-7) subfamilies. The KDM1 subfamily only accepts mono- and dimethylated lysines as substrates.

The available evidence is that lysine is one of, if not the, most diversely and extensively modified component monomer of any biological polymer [14], [15]. Lysine residues can be altered by modifications including acetylation, crotonylation, ubiquitination (multiple forms), hydroxylation (at least several types), and by *N*^ε^-methylation. Lysine and arginine residues can each exist in four biologically identified methylation states (unmodified, mono-, di- and tri-*N*^ε^-methylation for lysine and unmodified, mono-, di-(symmetric)- and di-(asymmetric)- methylation for arginine) (Fig. 1A). The possibility of combinations of modifications both on the same residue and on other histone residues enables an enormity of potential complexity that is proposed to be a central factor in the regulation of gene expression. Defining how the combinations of post-oligomerisation modifications to chromatin, and associated other variables (e.g. association of binding partner proteins) regulate context-dependent transcription of individual open reading frames is a major current biological challenge. Substantial difficulties arise in epigenetic regulation, including with inhibition, due to the context-dependent nature of effects, linked, at least in higher organisms, to redundancy and adaptation. The precise mechanisms of how *N*-methylation and other modifications regulate the transcription of individual open reading frames are complex and may take a long time, possibly decades, to be unravelled in detailed chemical terms. Whilst such an understanding is desirable, one would hope that it is not essential for therapeutic benefit to occur based on existing, or soon to be acquired, knowledge. Many enzymes catalysing chromatin modifications and their reversal, e.g. methylation/demethylation, have been identified; along with non-catalytic binding proteins, some are being pursued as therapeutic targets. Currently, the main therapeutic focus regarding methyltransferases and demethylases is cancer, but in the longer term the complexity of transcriptional regulation, and in particular methylation, suggests that the possibilities for selective treatment of many diseases involving transcriptional regulation are very substantial.

Histone methylation is catalysed by *S*-adenosylmethionine- (SAM) dependent histone lysine methyltransferases (KMTs) and protein arginine methyltransferases (PRMTs). Over-expression as well as both activating and inactivating mutations of many KMTs correlate with cancer [16]. In several acute leukaemias, the mixed lineage leukaemia (*MLL*) gene undergoes chromosomal translocation resulting in an oncogenic MLL fusion protein, which recruits DOT1L, an H3K79 KMT, causing aberrant methylation and transcription of leukaemeogenic genes [17], [18]. EZH2, a human H3K27 KMT which is a catalytic component of the PRC2 complex, is an oncogene overexpressed in many cancers and is associated with poor prognosis in breast and prostate cancers [19], [20], [21]. Other KMTs, such as the Nuclear Binding SET Domain protein (NSD1), SUV39H1 and SUV39H2, are also linked to cancers (see Ref. [16] for review). A DOT1L KMT inhibitor is in clinical development for *MLL*-rearranged leukaemia [22]. A KMT EZH2 inhibitor [23] is being targeted at non-Hodgkin lymphoma [24]. Together, with the clinical use of DNA methyltransferase inhibitors [25], these studies validate the modulation of chromatin methylation status for cancer therapy.

Over 20 human histone *N*^ε^-methylated lysine residue demethylases (KDMs) have been identified, with many exhibiting dysregulated expression patterns in disease [26], [27]. As with the KMTs, emerging evidence suggests that some KDMs are required for tumour growth. Consequently, KDMs have emerged as interesting new targets for therapeutic intervention. Demethylation of mono-*N*-methylated arginine residues on histones is proposed to be catalysed by the peptidyl arginine deiminases (PADIs) which catalyse hydrolysis to give citrulline and methylamine [28], [29]. Although there are reports of a ‘direct’ *N*-methylated arginine residue demethylase [30], [31], i.e. JMJD6, these are controversial. Subsequent work has revealed that JMJD6 is a lysine C-5 hydroxylase acting on arginine-rich regions of splicing regulatory proteins [32], [33]. Thus, at present there is no definitive evidence for direct reversal of arginine *N*-methylation.

KDMs are classified into two subfamilies depending on their sequence homologies and catalytic mechanisms. Members of the first identified subfamily, the KDM1 subfamily (or lysine-specific demethylases, LSDs), are related to the well-characterised monoamine oxidases (MAOs) and utilise the cosubstrate flavin adenine dinucleotide (FAD) during catalysis of demethylation; methyl group oxidation is proposed to proceed via hydride transfer from the *N^ε^*-methyl group onto FAD, forming an imine which is unstable to hydrolysis (Fig. 1B) [26]. For chemical reasons this mechanism cannot apply to quaternary amines, hence the KDM1 subfamily likely does not act on *N*-trimethylated lysine residues. KDM1A, the most studied KDM1 enzyme, catalyses removal of methyl groups from mono- and dimethylated lysine residues at H3K4 and, potentially, H3K9, while KDM1B catalyses demethylation specifically at H3K4 [26]. KDM1A is reported to be oncogenic and/or overexpressed in cancers including leukaemias and solid tumours [34], [35], [36], [37]. Notably, KDM1A is specific for H3K4me1/me2 when a component of the CoREST corepressor complex [38], but *in vivo* work has suggested that the presence of the androgen receptor (AR) may change its specificity from H3K4 to H3K9 [39]. Thus, KDM1A might potentially act either as a transcriptional corepressor or coactivator depending on its binding partners and substrates. This example nicely illustrates the importance of why a detailed biological understanding of context-dependent roles will be useful in interpreting the pharmacological effects of KDM inhibitors.

The JmjC domain-containing KDMs (KDM2-7 subfamilies; JmjC KDMs) represent the larger KDM class, comprising about 20 human enzymes which are grouped into five subfamilies (KDM2/7, KDM3, KDM4, KDM5, and KDM6) [40]. They can catalyse the demethylation of mono-, di- and trimethylated lysines at multiple sites, using 2OG and dioxygen as cosubstrates and Fe(II) as a cofactor [26]. The JmjC KDMs are over-expressed in multiple types of cancer cells [34], [37]. Some JmjC KDMs are implicated in neural development and/or function and are associated with conditions including X-linked mental retardation, autism and midline defects [41], [42], [43], [44]. The JmjC KDMs are related to *N*-methyl DNA and RNA demethylases (AlkB homologues and the fat mass and obesity-related protein, FTO) and other nucleic acid oxygenases including the TET (ten eleven translocation) hydroxylases which catalyse oxidation of the methyl group of 5-methylcytosine bases to give C-5 hydroxymethyl-, formyl, and carboxycytosine [45]. There is evidence that JmjC KDM-catalysed demethylation proceeds via the consensus mechanism for 2OG oxygenase catalysis: following sequential active site binding of 2OG, substrate, then dioxygen, oxidative decarboxylation occurs to give succinate, carbon dioxide and an Fe(IV)-oxo intermediate, which reacts with the *N^ε^*-methyl group, either via a radical rebound or direct insertion process to form an unstable hemiaminal [26]. Subsequent fragmentation produces the demethylated lysine and formaldehyde (Fig. 1B) [46], [47]. Thus, the overall reactions of the two KDM subfamilies are closely related; however, the JmjC KDMs are capable of catalysing demethylation of trimethylated lysines because their mechanism does not require imine formation.

The catalytic domain of the KDM1 subfamily, the amine oxidase-like (AOL) domain, is related to the large superfamily of monoamine oxidases [48], with MAO-A and MAO-B being the closest homologues [49]. MAOs catalyse the oxidation of monoamine neurotransmitters, such as serotonin and dopamine. Similarly, the catalytic domains of JmjC KDMs, the JmjC domains, are also part of the large superfamily of 2OG-dependent oxygenases, which has approximately 60 human members [50]. In addition to the roles of the JmjC KDMs in epigenetic regulation, other 2OG oxygenases play key roles in human biology, including in fatty acid metabolism, collagen biosynthesis, protein biosynthesis, nucleic acid repair/modification and hypoxic response [50].

**Fig. 2:**
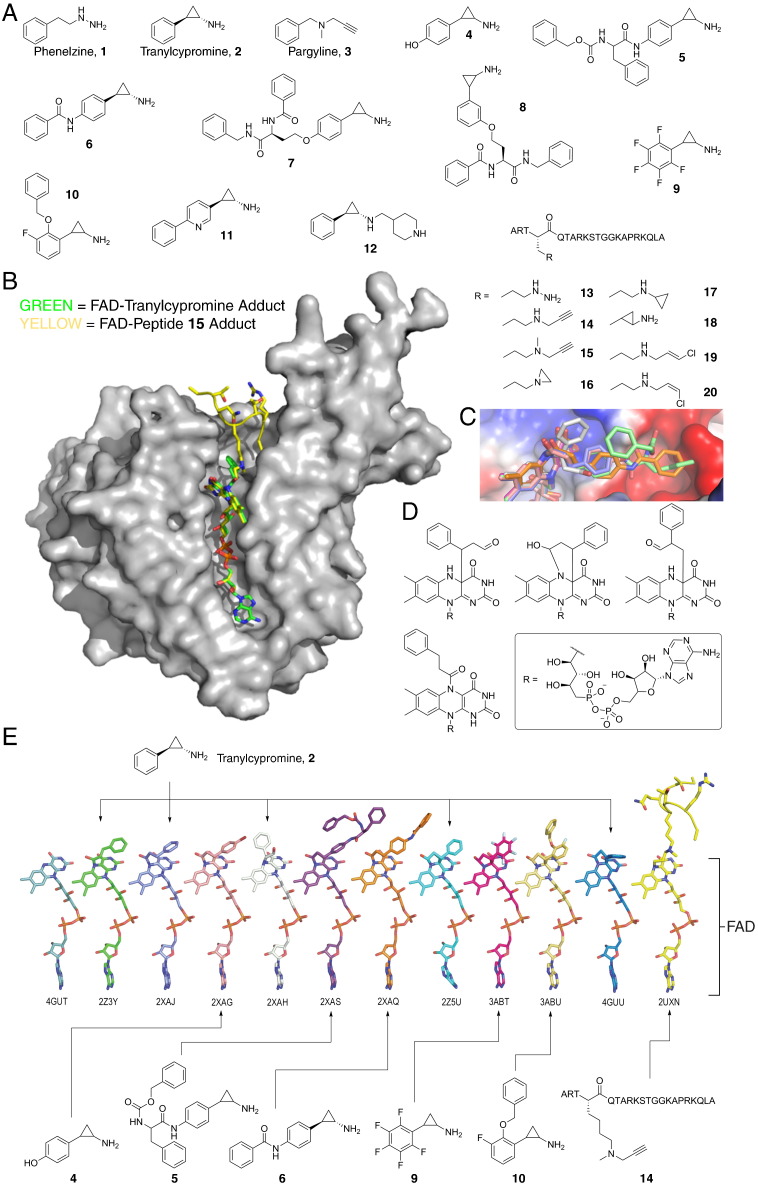
Mechanism-based inhibitors of the KDM1 subfamily. (A) Structures of representative mechanism-based KDM1 inhibitors. The MAO inhibitors phenelzine **1**, tranylcypromine **2** and pargyline **3** were among the first reported inhibitors of KDM1, which led to the development of KDM1-selective analogues. (B) Views from X-ray crystal structures of tranylcypromine (**2**) and peptidic inhibitor (**14**) cross-linked to FAD in the active site of KDM1A (PDB IDs: 2Z3Y[63] and 2UXN[155] respectively). Views from both structures are overlaid. (C) Views from crystal structures of tranylcypromine analogues (FAD adducts) in the active site of KDM1A (PDB IDs: 2XAJ, 2XAG, 2XAH, 2XAS and 2XAQ2XAS2XAQ) [61]. The analogues protrude into the substrate binding pocket. (D) Proposed structures of adducts formed by reaction of tranylcypromine with FAD in the active site of KDM1s. There is evidence for three of the structures from crystallographic analyses (see section E). (E) Structures of FAD adducts of mechanism-based inhibitors bound in the active sites of KDM1s (views of structures are from PDB IDs: 2Z3Y[63], 2XAJ[61], 2XAG[61], 2XAH[61], 2XAS[61], 2XAQ[61], 2Z5U[63], 3ABT[66], 3ABU, 4GUU[156] and 2UXN[155]).

Pioneering inhibitor development strategies for both KDM subfamilies initially focused on the testing of generic mechanism-related inhibitors for each family of enzymes. For example, the first generation KDM1 inhibitor, tranylcypromine (**2**, Fig. 2), is an FDA-approved MAO inhibitor that is used to treat mood and anxiety disorders [51]. Broad-spectrum 2OG oxygenase inhibitors, such as the 2OG mimetics *N*-oxalylglycine (NOG, **32**, Fig. 4) and pyridine-2,4-dicarboxylate (2,4-PDCA, **47**, Fig. 4), are also JmjC KDM inhibitors [52], [53]. Given the structural similarity of KDMs and the conservation of active site features with multiple homologous enzymes, one of the major challenges for future KDM inhibitor discovery is to develop inhibitors selective for particular (sets of) KDMs. Although beyond the scope of this review, it is also important to note that the activities of most, if not all KDMs, are likely directed by binding domains contiguous with the ‘catalytic’ JmjC domains (e.g. the KDM4 enzymes contain Tudor and PHD domains in addition to the JmjC domain) or by non-covalent interactions with targeting proteins (see above for KDM1A); these non-catalytic domains are also of therapeutic interest in isolation and in combination with KDM inhibitors. KDM inhibitors are required for target validation and for probing biological function. Below, we describe recent advances in the development of inhibitors for the KDM1 and JmjC KDM subfamilies, and discuss future strategies for KDM inhibitor discovery.

**Fig. 3:**
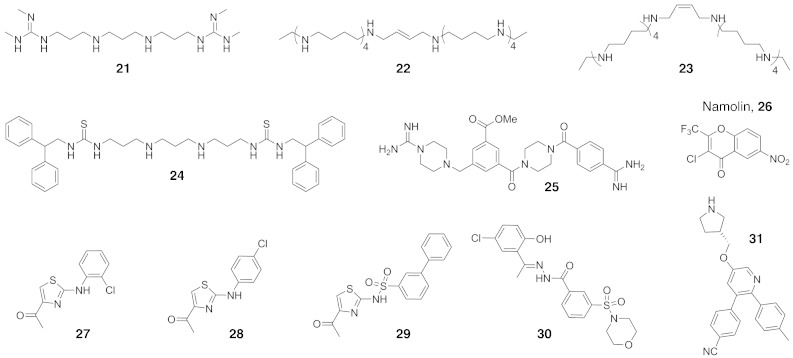
Structures of representative non-tranylcypromine-based KDM1 subfamily inhibitors. Bisguanidines, bisbiguanides, polyamines and (thio)urea-containing compounds (for examples see **21**–**24**) have all been shown to inhibit KDM1 activity in vitro, with some showing effects in cells. A detailed mechanistic/structural understanding of KDM1 inhibition by these compounds is presently not available. Recently, a series of other inhibitors of the KDM1 subfamily have been identified, including Namolin (**26**), aminothiazoles (**27**–**29**), benzohydrazides (**30**) and a tricyclic pyridine (**31**).

**Fig. 4:**
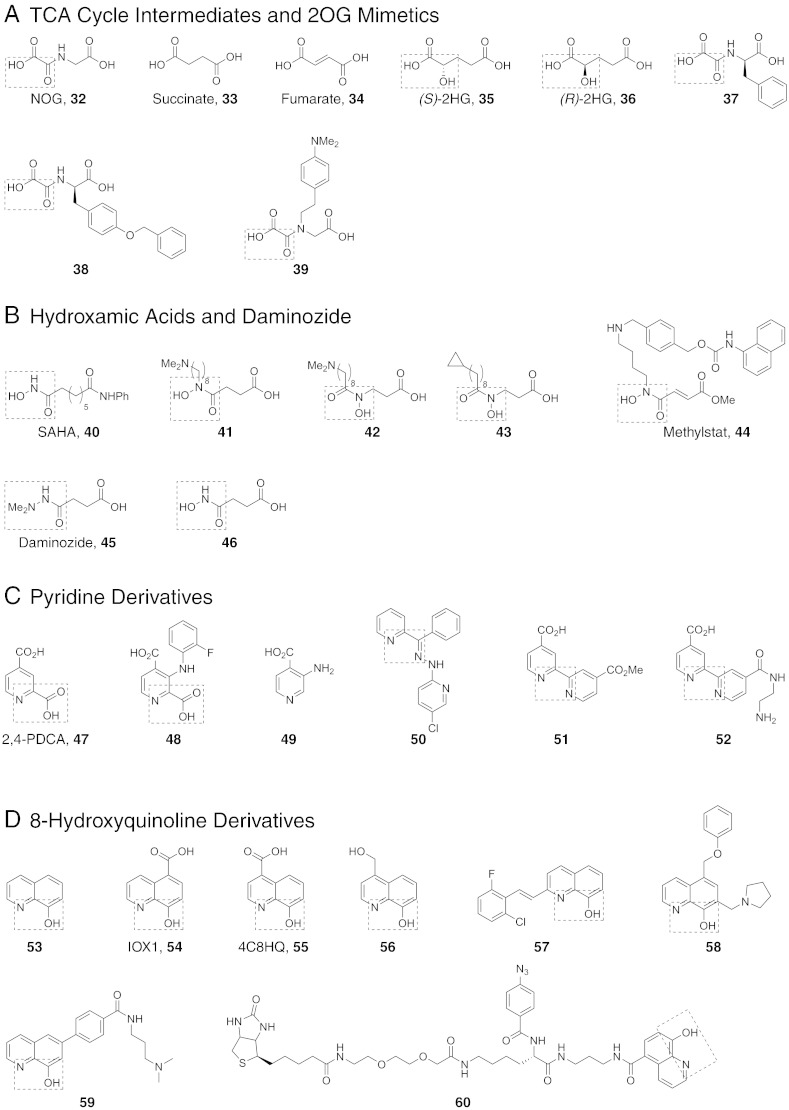
Structures of representative iron-chelating inhibitors of the JmjC KDMs. (A) Structures of tricarboxylic acid (TCA) cycle intermediates and 2OG mimetics. 2OG is a co-substrate of the 2OG oxygenases. Some 2OG oxygenases are inhibited by succinate (**33**, a co-product of catalysis), fumarate (**34**), and 2-hydroxyglutarate (**35** and **36**). Levels of these small molecules can be substantially increased in some tumour cells. *N*-Oxalylglycine (NOG, **32**) is a close isostere of 2OG. C-α derivatisation of NOG can confer selectivity for different 2OG oxygenase subfamilies. (B) Structures of hydroxamic acid-containing inhibitors and Daminozide (**45**). Hydroxamic acids are established metal chelators and with appropriate functionalisation can be potent and selective 2OG oxygenase inhibitors. Examples include the JmjC KDM inhibitor Methylstat (**44**), and SAHA (**40**), which is a clinically used inhibitor of histone deacetylases that also inhibits JmjC KDMs in vitro. Daminozide (**45**), a small achiral hydrazide, inhibits the KDM2/7 subfamily of JmjC KDMs. (C) Examples of pyridine-based KDM inhibitors. Pyridine-2,4-dicarboxylic acid (2,4-PDCA, **47**) is a broad-spectrum 2OG oxygenase inhibitor that chelates active site-bound iron via its pyridyl nitrogen and 2-carboxylate. KDM4-selective derivatives of 2,4-PDCA have been prepared via substitution at the 3-position (e.g. **48**). Recently, pyridine-containing fragments without a 2-carboxylate group (e.g. **49**) have also been reported to inhibit JmjC KDMs via monodentate iron chelation. Other pyridine-containing inhibitors include bipyridyl compounds (e.g. **51** and **52**), which chelate iron via both pyridyl nitrogens, and the pyridylhydrazone **50**, which was recently identified from a high-throughput screen. (D) Structures of 8-hydroxyquinoline derivatives. 8-Hydroxyquinolines chelate iron in a bidentate manner via their pyridyl nitrogen and phenolic oxygen atoms. 5-Carboxy-8-hydroxyquinoline (IOX1, **54**), which was identified from a high-throughput screen against KDM4E, is a broad-spectrum 2OG oxygenase inhibitor that exhibits moderate selectivity for some JmjC KDM subfamilies (KDM2/7, KDM3, KDM4, KDM6). Substitution at the 2-, 4-, 5- and 7- positions of the 8-hydroxyquinoline ring has resulted in improved selectivity for KDMs (e.g. **56**-**59**). Crystallographic studies with KDM4A, KDM6B and the HIF asparaginyl hydroxylase FIH reveal that IOX1 can induce translocation of the active-site iron (see Fig. 5, Fig. 7). Such metal movement is not observed for 4-carboxy-8-hydroxyquinoline (4C8HQ, **55**) binding to KDM4A (Fig. 5).

## 2 KDM1 inhibitors

### 2.1 Tranylcypromine and analogues

The known amine oxidase inhibitors phenelzine (**1**, Fig. 2A) [54], tranylcypromine (PCPA) (**2**, Fig. 2A) [54], [55], and pargyline (**3**, Fig. 2A) [39] were amongst the first compounds reported to inhibit KDM1 catalysis. Although subsequent studies with pargyline did not reveal KDM1 inhibition [54], [56], both the hydrazine derivative phenelzine **1** and cyclopropane derivative tranylcypromine **2** were subsequently found to inhibit KDM1A both in vitro and in cell-based experiments, with inhibitory potencies similar to those observed for these compounds against amine oxidases [54], [57]. Structural and mechanistic studies with MAOs have shown that phenelzine **1** and tranylcypromine **2** react with the co-substrate FAD via their hydrazine and cyclopropyl groups respectively, forming covalent adducts within the active site [58], [59], [60]. Analogous ‘suicide’ inactivation has also been reported for KDM1A inhibition by tranylcypromine **2** (Fig. 2) [55], [61], [62], [63].

Subsequent studies, which were partly informed by crystallographic analyses, have utilised tranylcypromine **2** as a scaffold for the development of more selective cyclopropane-based KDM1 inhibitors (for representative structures of analogues, see Fig. 2A) [61], [64], [65], [66], [67], [68], [69], [70], [71]. To date, a number of tranylcypromine analogues, modified on both the aromatic ring and amino groups, have been tested for KDM1 activity, resulting in the generation of inhibitors which display both improved inhibitory potency against KDM1A and, in some cases, improved selectivity.

Improvements in both potency and selectivity were observed for compounds substituted on the aromatic ring of tranylcypromine [61], [65], [66], [70], [71]; crystallographic analyses reveal that these groups likely protrude into the methylated lysyl binding pocket, forming favourable hydrogen-bonding and hydrophobic interactions (Fig. 2) [61], [66]. Some of the tranylcypromine derivatives inhibit cell growth in cancer cell lines including murine acute promyelocytic leukaemia blasts [61], [65], [72]. In addition, a number of compounds substituted at the primary amino group display improved activity relative to tranylcypromine **2** and are selective for the KDM1 family over related amine oxidases [67], [68], [69]. Some of these compounds are brain-penetrating [67]. Promising preclinical data for treatment of acute myeloid leukaemia (AML) by targeting KDM1A with tranylcypromine **2** have provided target validation evidence. A KDM1A inhibitor has been recently described which, it is reported, shows both unprecedented in vitro potency (IC_50_ at 20 nM) and selectivity over all analysed amine oxidases including KDM1B. This compound (ORY-1001) is projected to enter clinical trials in 2014 [73].

### 2.2 Substrate mimetics

The preference of the KDM1 subfamily for protein substrates, as well as their susceptibility to mechanism-based inhibitors (e.g. tranylcypromine **2**), has led to the development of a series of peptides containing reactive groups selective for KDM1 over MAOs. Peptide fragments of H3 were synthesised with K4 lysine analogues [57], [74] and modified to contain reactive groups (e.g. cyclopropyl, propargyl) intended to react with the FAD cofactor of the KDM1 enzymes. A number of the peptides inhibited KDM1A activity in vitro, with a hydrazine-derivatised peptide showing the most potent activity (**13**, Fig. 2C, *K_i_* = 4.4 nM). Mechanistic and crystallographic studies with the propargyl group-containing peptides (**14** and **15**, Fig. 2) revealed that inhibition by these peptides is indeed induced, at least in part, through reaction with FAD [62], [75] (a similar mechanism for inhibition by analogous peptides has also been proposed) [57].

### 2.3 Polyamines

Polyamines are known MAO inhibitors which also inhibit KDM1 [76], [77]. An early screen of bisguanidines and bisbiguanides identified non-competitive inhibitors of KDM1A with low-micromolar IC_50_ values (for representative structures, see Fig. 3) [78]. Two of the compounds were tested in cell models, resulting in increased expression of tumour suppressor genes as well as fluctuations in levels of H3K4me2 (increase), H3K9ac (increase), H3K4me1 (decrease) and H3K9me2 (decrease) marks. Later studies with a series of polyamines (octamines and decamines) also revealed inhibition of recombinant KDM1A and similar effects in cells (Fig. 3) [79]. Further derivatives incorporating bisurea and bisthiourea groups also exhibited KDM1A activity, with two compounds inhibiting growth of non-small cell lung carcinoma cells (Fig. 3) [80]. Two recent patents also reveal KDM1A inhibition by amidine- and dithiocarbamate-containing compounds [81], [82]. However, more detailed mechanistic, selectivity and cell-based studies are required for the ‘polyamine’ inhibitor class. In particular, it is of interest to determine to what extent the cell-based observations result from selective KDM1 subfamily inhibition.

### 2.4 Other KDM1 inhibitors

Several types of reversible inhibitors of KDM1 have recently been reported. Namolin (**26**, Fig. 3), a cell-active, reversible inhibitor of KDM1A, was identified by screening against the closely related monoamine oxidases MAO-A and MAO-B [49]. Aminothiazoles have been identified as weak reversible inhibitors (compounds **27** and **28**, IC_50_ at 249 and 437 μM respectively, Fig. 3) of KDM1A following a fragment-based screen [83]. The initial hits were developed to increase potency (**29**, lowest IC_50_ at 7 μM, Fig. 3); however cellular assays using CD86 expression as a marker for KDM1A inhibition in THP1 cells [84] indicated that these compounds are not cell-active (EC_50_ > 50 μM).

A structure-based virtual screen, followed by computational analyses and biochemical screening, led to the identification of an *N*′-(1-phenylethylidene)-benzohydrazide series as KDM1A inhibitors. The optimised compound **30** was found to be highly potent (K_i_ = 31 nM) and selective over monoamine oxidases (MAO-A and MAO-B > 300 μM), to inhibit cell proliferation of several cancer cell lines, and to increase H3K4me2 levels in VCaP cells in a dose-dependent manner [85]. Recently, GSK354, a novel potent (IC_50_ ≤ 100 nM), highly selective (MAO IC_50_ ≥ 200 μM) and cell-active inhibitor (**31**, IC_50_ = 1.4 μM, Fig. 3) of KDM1A has been declared as a chemical probe suitable for use in biological function studies [86].

## 3 JmjC KDM inhibitors

### 3.1 Overview

The 2OG oxygenases were originally identified as playing roles in collagen biosynthesis comprising prolyl and lysyl hydroxylations. Prolyl-4-hydroxylation stabilises the collagen triple helix and consequently, in pioneering inhibition work, collagen prolyl-4-hydroxylases were targeted for fibrotic diseases [87], [88]. These studies led to the identification of various compounds that compete with 2OG and coordinate to the active site ferrous iron [89]. Prior to the identification of the JmjC KDMs, 2OG oxygenases were found to play key roles in the physiological response to limiting oxygen availability via the post-translational prolyl and asparaginyl hydroxylation of the hypoxia-inducible transcription factor (HIF) [90]. Whereas the HIF prolyl hydroxylases (PHD or EGLN enzymes) are related to the collagen prolyl hydroxylase subfamily of 2OG oxygenases, HIF asparaginyl hydroxylation is catalysed by a JmjC-type enzyme (factor inhibiting HIF, FIH) but one which catalyses a stable hydroxylation modification rather than demethylation via hydroxylation [90]. Some of the inhibitors developed/applied to the collagen and HIF hydroxylases, such as *N*-oxalylglycine (NOG, **32**, Fig. 4), have been found to be useful both as broad-spectrum 2OG oxygenase inhibitors, including for the KDMs, and as templates for development by functionalisation into more specific inhibitors [89].

There is now a considerable amount of structural information available for 2OG oxygenases [91], [92]. The crystal structures have revealed substantially conserved Fe(II) and 2OG binding sites; however, differences in Fe(II) and 2OG binding sites are subfamily-characteristic and can be exploited for the development of selective inhibitors [92], [93]. As may be anticipated from the diversity of substrate types for 2OG oxygenases – proteins, nucleic acids, small molecules and lipids – there are greater variations in the substrate binding elements, which separately, or in conjunction with Fe(II) binding, can be exploited for the development of selective inhibitors. However, approaches involving solely mimicking the binding of histone substrates to the JmjC KDMs raise the possibility of selectivity issues relating to non-2OG oxygenase proteins that bind the same/similar histone sequences. Further, it is important to note that 2OG oxygenase catalysis involves an ordered sequential mechanism involving conformational changes that are not yet fully defined and probably not (completely) accessible by crystallographic studies. For example, recent studies with inhibitors have demonstrated that some compounds can induce movement (> 1.0 Å) of the metal within the active site [53], [94]. Thus, structural analyses by solution phase techniques (e.g. NMR) are of importance in the field [95], [96].

It is also worth mentioning that inhibition of 2OG oxygenases by endogenous small molecules is of pathophysiological relevance. Mutations to tricarboxylic acid (TCA) cycle enzymes are common in tumours and can result in very substantial increases in the concentrations of succinate (**33**, Fig. 4), fumarate (**34**, Fig. 4), or 2-hydroxyglutarate ((*S*/*R*)-2HG, **35** and **36**, Figs. 4/5) [97], [98], [99]. The formation of 2HG is of particular biochemical interest — ‘gain of function’ mutations cause isocitrate dehydrogenase (IDH) 1 and 2 to convert 2OG into 2HG, as well as produce 2OG from isocitrate [100]. The increases in TCA intermediate/2HG levels are proposed to be pro-oncogenic, at least in part due to inhibition of 2OG oxygenases involved in transcriptional regulation. Succinate [101], [102], which is a co-product of 2OG oxygenase catalysis, fumarate [103], [104], and 2HG all inhibit 2OG oxygenases, though rather weakly (in the μM to mM range for KDM2A, KDM4A, KDM4C and KDM5B, as shown with isolated proteins and in cells [105], [106]), via competition with 2OG [103], [107], though more complex mechanisms are possible. It is also notable that succinate release is rate-limiting for some 2OG oxygenases [108] and that high levels of the cosubstrate 2OG can be inhibitory [109]. Further, in assessing the potential relevance of 2OG oxygenase inhibition by endogenous 2OG competitors, consideration should be given to the 2OG/inhibitor concentrations in cells, enzyme concentration, and 2OG K_M_/K_D_ values; the latter can vary substantially (> 100-fold) [110]. Ester derivatives of TCA intermediates, 2HG and 2OG have been used as prodrugs in cellular functional studies because the parent di-acids have poor cell penetration [111].

**Fig. 5:**
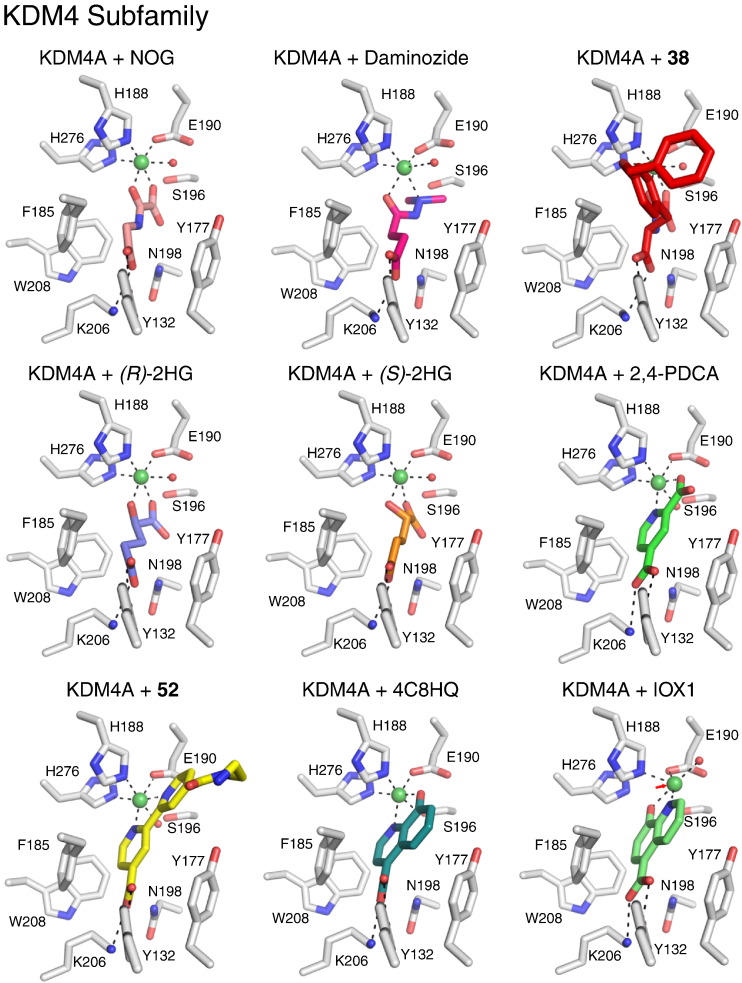
Crystal structures of inhibitors bound to KDM4A. KDM4 inhibitor structures reported to date are for bidentate iron chelators. One chelating group from the inhibitor coordinates opposite to the iron-binding glutamate residue (Glu190 in KDM4A); however, the second chelating group may coordinate either opposite the first iron-binding histidine (His188 in KDM4A), as observed for 2OG chelation (and for NOG, see top left), or opposite the second histidine (His276 in KDM4A), as observed for 2,4-PDCA (see middle right). The preference of the two binding modes is likely determined by steric factors. Binding of IOX1 in the active site of KDM4A induces translocation of the active site metal (red arrow, bottom right). In all structures, the active site iron is substituted for nickel. PDB IDs: 2OQ6[121], 4AI9[124], 2WWJ[52], 2YBK[105], 2YBS[105], 2VD7[104], 3PDQ[134], 4BIS[53] and 3NJY[135].

In the following sections we describe studies on various types of JmjC KDM inhibitors — our objective is not to be comprehensive (especially with respect to the patent literature) but to illustrate different types of inhibitors developed to date and make general points. Reflecting current reports, our focus is on active site-directed inhibitors, but (evidently) there is also potential for the inhibition of auxiliary domains, and by targeting the non-active site regions in the core JmjC domain. The latter approach is exemplified by compounds that act on the zinc-binding domain of KDM4 [112]. It is also notable that recent work has focused on inhibition of the JmjC KDMs, with fewer reports to date on the nucleic acid oxygenases such as FTO and the TET enzymes [113], [114], [115].

### 3.2 *N*-Oxalyl amino acid-based JmjC KDM inhibitors

*N*-Oxalylglycine (NOG, **32**, Fig. 4) is a closely related analogue of 2OG wherein the C-3 methylene group is replaced with an NH group to give an *N*-oxalyl amide derivative that likely stalls the catalytic reaction by hindering oxygen binding to the active site iron. Although first described as a prolyl hydroxylase (PHD) inhibitor, NOG is a broad-spectrum inhibitor of many 2OG oxygenases [116], which, following initial work with KDM4E [104], has been shown to inhibit all tested JmjC KDMs [53]. Multiple crystal structures for 2OG oxygenases complexed with NOG have been reported (e.g. for KDM4A, KDM6A, PHF8; see Fig. 5, Fig. 11); the structures reveal that NOG binds to the active site metal (iron or an iron surrogate) via its C-2 oxo and C-1 carboxylate groups, with its glycine carboxylate occupying the same position as the C-5 carboxylate of 2OG. Although NOG is a relatively broad-spectrum inhibitor, it should be noted that the potency of 2OG oxygenase inhibition by NOG does vary substantially [53]. The cell-penetrating dimethyl ester form of NOG, dimethyl *N*-oxalylglycine (DMOG), has found widespread applications as a hypoxia mimetic [117], [118] because it inhibits both HIF prolyl and asparaginyl hydroxylases resulting in upregulation and activation respectively of the HIF system [119]. It is possible that some of the observed cellular effects of DMOG result from JmjC KDM, or other 2OG oxygenase, inhibition [53].

**Fig. 6:**
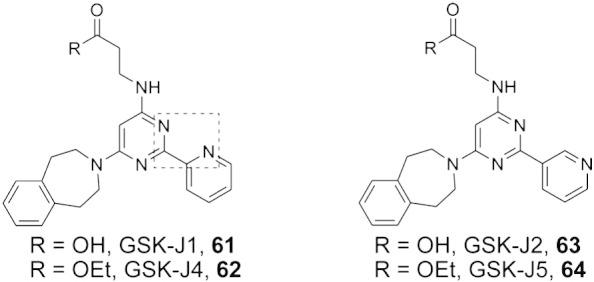
Structures of the KDM6 subfamily inhibitor GSK-J1 and derivatives. GSK-J1 (**61**) is an iron chelator, which induces metal movement within the active site of KDM6B (for a view of a crystal structure of GSK-J1 bound in the active site of KDM6B, see Fig. 11). The ethyl ester prodrug (GSK-J4, **62**) is active in cells, whereas the prodrug form of the inactive analogue GSK-J2 (**63**) shows no cellular effects (GSK-J5, **64**).

**Fig. 7:**
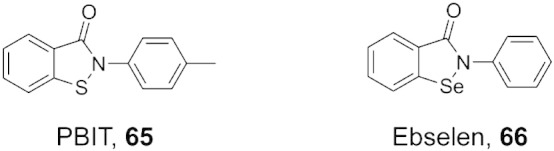
Structures of 2-4(4-methylphenyl)-1,2-benzisothiazol-3(2H)-one (PBIT) and ebselen. PBIT (**65**) and ebselen (**66**) inhibit both the KDM4 and KDM5 subfamilies of KDMs, probably via removal of the enzyme-bound zinc.

**Fig. 8:**
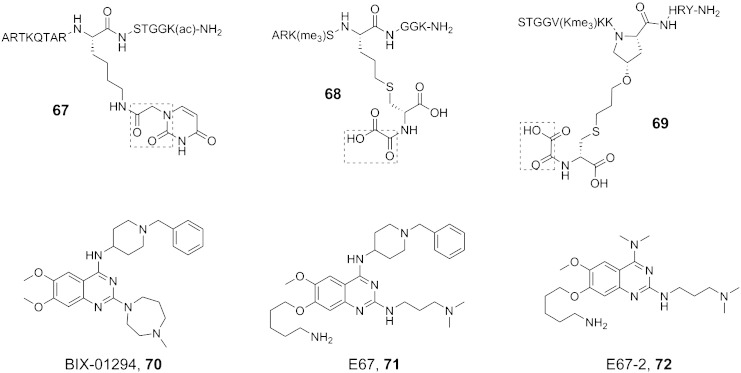
Structures of histone substrate-competitive JmjC KDM inhibitors. Compounds **67**-**69** comprise both iron-chelating groups and histone fragments to enable potency and selectivity for JmjC KDMs. The lysine methyltransferase inhibitor BIX-01294 (**70**) also inhibits JmjC KDMs; development of BIX-01294 analogues has resulted in JmjC KDM-specific inhibitors (**71** and **72**). Crystallographic studies of **71** with KDM7A suggest that the compound may compete with the histone substrate for binding (Fig. 11).

**Fig. 9:**
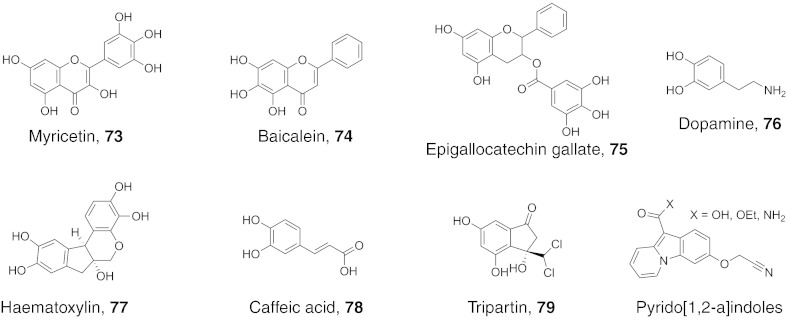
Structures of flavonoid, catechol, tripartin, and pyrido[1,2-a]indole inhibitors of JmjC KDMs. Numerous flavonoid and catechol compounds have been identified as JmjC KDM inhibitors from high-throughput screening, including myricetin (**73**) and dopamine (**76**). Precise mode(s) of inhibition, however, are unclear. Tripartin (**79**) is an indanone-based natural product that was shown to inhibit KDM4. Pyrido[1,2-a]indoles have been recently reported to inhibit KDM4C via unknown mechanism(s).

**Fig. 10:**
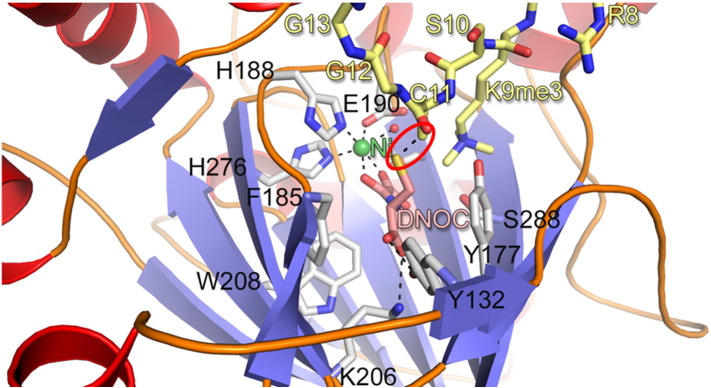
View from a crystal structure of KDM4A with bound peptide substrate analogue (sequence ARK(me3)SCGGK, yellow) and *N*-oxalyl-D-cysteine (DNOC, pink). The peptide and DNOC form a disulfide linkage within the active site (dashed line, circled). Replacing the disulfide bond with a sulfide led to the development of a stable analogue exhibiting potent and selective inhibition for the KDM4 subfamily (**68**, Fig. 8). PDB ID: 3U4S[141].

**Fig. 11:**
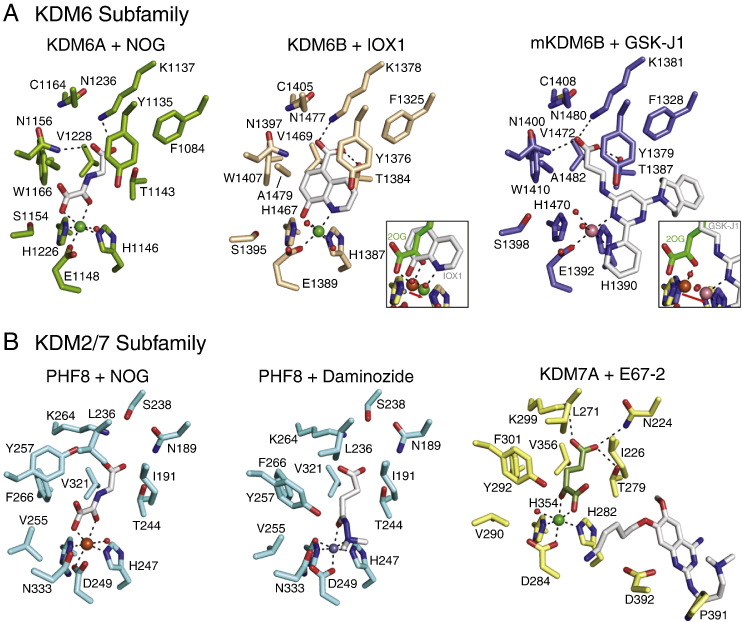
Views from crystal structures of inhibitors bound to the KDM6 and KDM2/7 subfamilies. As observed in inhibitor structures with KDM4A, bidentate iron chelators may adopt one of two coordination modes around the iron, with one iron-binding group positioned opposite to the iron-binding carboxylate residue (Glu1148 in KDM6A, Glu1389 in KDM6B, Asp249 in PHF8 and Asp284 in KDM7A) and the other opposite to one of the iron-binding histidines. Both IOX1 (**54**, Fig. 4) and GSK-J1 (**61**, Fig. 7) induce metal translocation in KDM6B (note: the crystal structure of GSK-J1 binding was solved using mouse KDM6B). E67 (71, an analogue of the lysine methyltransferase inhibitor BIX-01294, 70, Fig. 8) binds away from the iron binding site in KDM7A, suggesting no metal chelation. PDB IDs: KDM6A + NOG, 3AVS (metal = nickel) [157]; KDM6B + IOX1, 2XXZ (metal = nickel) [53]; mKDM6B + GSK-J1, 4ASK (metal = cobalt) [94]; PHF8 + NOG, 3KV4 (metal = iron) [158]; PHF8 + Daminozide, 4DO0 (metal = zinc) [124]; KDM7A + E67, 3U78 (metal = nickel) [142].

The potential for selective inhibition of the JmjC subfamily was demonstrated by pioneering studies on inhibition of the HIF hydroxylases. Comparison of PHD2 and FIH crystal structures led to the proposal that C-alpha derivatisation of NOG may lead to FIH-selective inhibitors [120]. Indeed the NOG derivative *N*-oxalyl-d-phenylalanine (**37**, Fig. 4) is selective for FIH over the PHDs; the dimethyl ester prodrug form is an active FIH inhibitor in cells [119]. The benzyl group of **37** occupies a hydrophobic binding pocket in FIH close to the Fe(II) binding site which is absent in the PHDs.

As with FIH, the KDM4 subfamily also possess a hydrophobic pocket close to the Fe(II) centre, which may be exploited for selective inhibition [121]. A combined approach involving structure-guided design coupled with disulfide-based dynamic combinatorial chemistry employing non-denaturing mass spectrometry (DC-MS) for screening was employed to identify disulfides that occupied this pocket, which is larger in the KDM4s compared to FIH. Subsequently, stable analogues of disulfides that were observed to bind relatively tightly by MS were prepared. It was found that substitution at the *para* position of the phenyl ring of *N*-oxalyl-d-tyrosine resulted in molecules that inhibit KDM4E in the micromolar range and which are selective over both FIH and the PHDs [52]. A crystal structure of KDM4A in complex with one of the potent inhibitors (**38**) was obtained (Fig. 5).

Analysis of the X-ray crystal structure of KDM4A in complex with NOG (occupying the 2OG binding site) and a trimethylated peptide (occupying the substrate pocket, PDB ID: 2OQ6) [121] led to the design and synthesis of NOG derivatives substituted with an alkyl-linked dimethylaniline group in order to mimic the interactions of the trimethylated peptide with the protein (Fig. 4) [122]. These derivatives maintain the inhibitory action of NOG against KDM4A, and exemplify the strategy of linking the 2OG and peptide substrate binding sites for JmjC KDM inhibition (for representative structures, see **39**, Fig. 4) [122].

### 3.3 Hydroxamic acid-based JmjC KDM inhibitors

In early screenings for templates that inhibit the JmjC KDMs, work with KDM4E identified hydroxamic acid inhibitors, including conformationally constrained 2OG analogues. Interestingly, amongst the hydroxamic acids found to inhibit KDM4E was the histone deacetylase (HDAC) inhibitor SAHA (Vorinostat, **40**, Fig. 4) [104]. This observation raises the possibility that some of the pharmacological effects of SAHA may result from inhibition of targets other than HDACs, including the JmjC KDMs.

The inhibitory activity of SAHA and the knowledge that hydroxamic acids are good metal chelators led to the design of inhibitors where the 2-oxoacid group of 2OG is replaced with a hydroxamic acid moiety [123], [124]. The resulting compounds containing the hydroxamic acid scaffold show good selectivity for the KDM4 family over the related HIF PHDs. Molecules where the hydroxamic acid scaffold is connected through an alkyl linker to a dimethylamino group were designed based on the KDM4A:NOG crystal structure (PDB ID: 2OQ6 [121]) [125]. The protein interaction of the protonated tertiary amine likely mimics those of the positively charged *N^ε^*-methylated lysine residue. A linker of eight carbon atoms was found to give optimum potency for KDM4A and KDM4C inhibition (**42**, Fig. 4). Replacing the dimethylamino group of 42 with a cyclopropane ring (43, Fig. 4) shifted the selectivity from the KDM4 subfamily to the KDM2/7 subfamily. It is proposed that the cyclopropane group occupies a hydrophobic pocket present in the KDM2/KDM7 subfamily.

The strategy of designing inhibitors capable of binding to both the 2OG and substrate binding pockets was applied in the discovery of Methylstat (**44**, Fig. 4), which contains a carboxyhydroxamic acid designed to occupy the 2OG binding pocket attached to a protonated lysine mimic through a hydrocarbon linker. Whilst the free acid inhibits KDM4C in the micromolar range, the methyl ester was found to be necessary for cell activity [126].

### 3.4 Daminozide

Daminozide (**45**, Fig. 4) is a (now largely discontinued) agrochemical, which inhibits 2OG oxygenases involved in gibberellin biosynthesis in plants [127]. Following screening of a set of potential 2OG analogues, Daminozide was found to also inhibit the KDM2/7 family enzymes (KDM2A (IC_50_ = 1.5 μM) and PHF8 (IC_50_ = 0.55 μM)) — somewhat unexpectedly given its small size. Some analogues of Daminozide, including a hydroxamic acid variant (**46**, Fig. 4), also revealed inhibition, albeit with poorer selectivity (and in some cases, poor compound stability). Daminozide is selective for the KDM2/7 subfamily over other members of the human JmjC KDMs. Crystallographic studies reveal Daminozide chelates the active site iron via its dimethylamino nitrogen lone pair and C-4 carbonyl group, with its C-1 carbonyl occupying the same site as the 2OG C-5 carboxylate (Figs. 5/11). This selectivity may be engendered by the more lipophilic region created by the Tyr257, Val255 and Ile191 residues adjacent to Fe(II) in the KDM2/7 subfamily compared to the more hydrophobic residues in the corresponding regions in other JmjC demethylases, as indicated by crystallographic studies [124].

### 3.5 Pyridine dicarboxylates and related JmjC KDM inhibitors

Pyridine dicarboxylates are more rigid 2OG analogues than NOG and the acyclic hydroxamic acids. Of the various pyridine-dicarboxylate regioisomers, pyridine-2,4-dicarboxylic acid (2,4-PDCA, **47**, Figs. 4/5/12) is the broadest-spectrum 2OG oxygenase inhibitor, and is an inhibitor of most JmjC KDMs. [104] It is used as dimethyl or diethylester prodrugs for cellular studies where it has been shown to inhibit JmjC KDM activity by antibody- and MS-based assays [128].

**Fig. 12:**
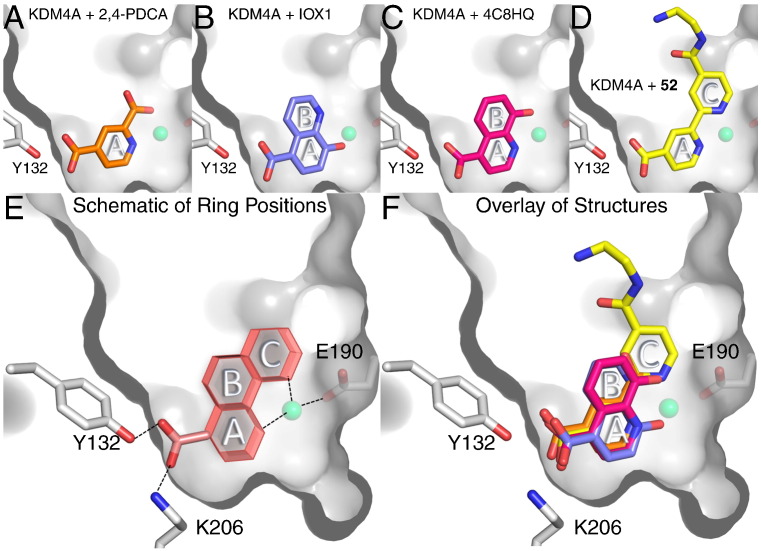
Analysis of heterocyclic iron chelators binding to JmjC KDMs. Overlays of reported structures of heterocyclic inhibitors bound to JmjC KDMs reveal preferential positioning of 2 aromatic groups within the active sites. In structures of KDM4A with bound 2,4-PDCA (section A), IOX1 (section B), 4C8HQ (section C) and the bipyridine compound **52** (section D), an aromatic group is positioned between the metal site and the 2OG C-5 carboxylate binding site defined by residues Tyr132 and Lys206 (ring position A, section E). In KDM4A, the position of this aromatic group is likely stabilised by π-π stacking interactions with Phe185. For bicyclic chelators (IOX1, 4C8HQ and **52**), the other aromatic groups are positioned in one of two discrete orientations (ring positions B or C, sections E and F). Despite IOX1 inducing translocation of the active site metal, the position of quinolyl bicyclic system is identical to that adopted by 4C8HQ, suggesting metal movement may be preferred to altered ring-positioning, at least in KDM4A.

The observation that the 2OG binding pocket is smaller in the HIF prolyl hydroxylases than the KDM4 demethylase subfamily led to the design of 2,4-PDCA derivatives substituted at the C-3 position to block binding in PHD2 whilst retaining potency against KDM4E. Notably, one of the most potent inhibitors was the 3-amino derivative **48** (Fig. 4) [129]. Although the 2-carboxy group in 2,4-PDCA and its derivatives is involved in binding to Fe(II) in the active site, 3-amino-4-carboxy pyridines lacking the 2-carboxy group (e.g. **49**, Fig. 4) have also been recently described as JmjC inhibitors [130]. The 4-carboxy group, which mimics the 2OG carboxylate, is also not essential for JmjC inhibition; screening of the National Cancer Institute's ‘Diversity Set’ library resulted in the identification of a pyridylhydrazone derivative as a micromolar to sub-micromolar inhibitor of KDM4A–E, KDM6B and KDM5A (**50**, Fig. 4) [131]. This compound (JIB-04) was also found to have an anti-proliferative effect in cancer cell lines and to inhibit tumour growth in mice. The *Z*-isomer was less efficacious in causing cell death, possibly because of reduced demethylase activity as a result of being unable to chelate Fe(II) in the active site in a bidentate manner.

### 3.6 Bipyridine and related JmjC KDM inhibitors

2,2′-Bipyridine and its derivatives are precedented 2OG oxygenase inhibitors including of the HIF PHDs [132], [133]. Screening efforts have shown that the carboxy derivative 4′-(methoxycarbonyl)-2,2′-bipyridine-4-carboxylic acid (**51**, Fig. 4) inhibits KDM4E in the micromolar range [104]. More potent analogues were discovered by replacing the methyl ester with a range of substituted amides. Optimisation of the amide substituent led to compound **52** (Fig. 4) which has 40-fold improved KDM4E activity (180 nM) compared to **51** [134]. Crystallographic studies demonstrated that **52** binds to KDM4A in the active site, chelating Fe(II) through the two pyridyl nitrogens (Figs. 5/12).

### 3.7 8-Hydroxyquinoline-based JmjC KDM inhibitors

A high-throughput screen led to the identification of a variety of 4-, 5-, and 7-substituted 8-hydroxyquinolines that are active against KDM4E [135]. The 5-substituted hit compounds were most active and were validated in a secondary assay against KDM4A and KDM4E. These compounds also showed activity against FIH and PHD2 although with somewhat weaker potency. The most potent compound, 5-carboxy-8-hydroxyquinoline (IOX1, **54**, Fig. 4) displayed an IC_50_ of 0.2 μM in vitro and was active in cells with an EC_50_ of 87 μM. IOX1 is a broad-spectrum 2OG oxygenase inhibitor, displaying sub-micromolar in vitro potency against KDM4A/C/D/E and KDM3A, micromolar IC_50_s against KDM6A, KDM2A, PHF8 and KDM5C, and high-micromolar activity against BBOX1 [53]. IOX1 chelates Fe(II) in the enzyme active site in a bidentate manner via its quinoline nitrogen and phenol oxygen atoms. The carboxylic acid at the 5-position interacts with Tyr132 and Lys206 in a way similar to the acyclic 2OG mimetic NOG (Figs. 5/11/12). The regioisomer of IOX1, 4-carboxy-8-hydroxyquinoline (4C8HQ, 55, Fig. 4) binds in an analogous way to IOX1 by chelating Fe(II) and interacting with Tyr132 and Lys206 (Figs. 5/12). However, in the case of IOX1 **54**, the position of the active-site metal is shifted relative to its position in the absence of inhibitor (as observed for a selective KDM6 inhibitor, see below) [53]. This iron translocation may be related to the difference in potency between IOX1 **54** and 4C8HQ **55**.

The activity of the 8HQ-based KDM4 inhibitor **59** (Fig. 4) has been reported to be related to suppression of initiation of infection by herpes simplex virus, suppression of propagation of infection to adjacent cells and suppression of reaction in latently infected sensory neurons via inhibition of the initiation stage [136]. These results suggest that chromatin remodelling via small-molecule modulation may be a viable strategy for antiviral therapy at an early stage of infection. More generally they suggest potential of ‘epigenetic’ therapies for treatment of infectious diseases.

Photoaffinity probes show promising utility for identification and quantification of proteins and mapping molecular interactions in cells [137]. Such a photoaffinity probe (**60**, Fig. 4) for 2OG oxygenases was developed by coupling IOX1 to a photoactivatable crosslinking group and biotin as an affinity-purification tag [138]. The resulting probe allowed the identification of KDM2A in HEK293T cells via photo-crosslinking and affinity purification coupled to MS analysis. In an effort to develop this methodology for profiling various 2OG oxygenases from different cell types, the Ugi four-component reaction was exploited to enable empirically guided optimisation of probes for specific tasks by enabling the modular assembly of probe scaffolds with a diverse set of photoreactive groups, detection handles and inhibitor attachment points [139].

### 3.8 A small-molecule probe for KDM6

The first small-molecule selective probe for the KDM6 subfamily (KDM6A and KDM6B) is GSK-J1 (**61**, Fig. 6), with its corresponding cell-active ethyl ester prodrug GSK-J4 (**62**, Fig. 6) [94]. This active site-binding inhibitor reduced the KDM6A- and KDM6B-mediated lipopolysaccharide-induced proinflammatory cytokine production by human primary macrophages. GSK-J1 chelates the active site Fe(II) and contains a propanoic acid side-chain that mimics 2OG side chain binding: like IOX1 (see above), GSK-J1 induces movement of the active site metal (Fig. 11). The negative control compound GSK-J2 (63, Fig. 6), is a regioisomer of the pyridine moiety of GSK-J1 which cannot engage in bidentate metal chelation and renders the molecule inactive (for the corresponding prodrug GSK-J5, see 64, Fig. 6). However, GSK-J4 has also been reported to inhibit the KDM5 subfamily in vitro (~ 20-fold less active relative to KDM6 inhibition) further highlighting the difficulties in achieving selectivity for this class of enzymes.

The benzisothiazol-3-one ring system is currently being pursued for KDM5 inhibitor discovery. 2-4(4-Methylphenyl)-1,2-benzisothiazol-3(2H)-one (PBIT, **65**, Fig. 7) was recently identified as an inhibitor of KDM5B (IC_50_ = 3 μM) through a high-throughput screen programme. Its analogue, Ebselen (**66**, Fig. 7), also exhibits similar inhibitory activity. PBIT inhibits KDM5A/B/C but does not inhibit KDM6A/B. While benzisothiazol-3-ones have also been shown to inhibit the KDM4 subfamily [112], [135], it is possible that selectivity may be achieved with further medicinal chemistry efforts. PBIT is cell-active and can inhibit transiently overexpressed KDM5B, thereby increasing global levels of H3K4me3. Although the mechanism of action of these compounds is unknown, it is possible that ejection of enzyme-bound zinc may be important for their activity (as observed for inhibition of KDM4A by Ebselen) [112].

### 3.9 JmjC KDM inhibitors targeting the substrate binding pocket

The JmjC KDMs have very distinct, sometimes overlapping, histone H3 substrate preferences. Emerging work is focusing on the use of histone peptide substrate mimetics as a strategy to achieve inhibitor selectivity for specific (sets of) JmjC KDMs. In one study, a systematic truncation analysis of histone H3K9me3/2 peptides and PHF8/KDM4A/C turnover demonstrated differential activities amongst the JmjC KDMs for the same H3 sequence [140]. The peptides were then converted to inhibitors by conjugating a promiscuous iron chelator motif, uracil, onto the K9 position of the truncated peptides, and the degree of peptide length imparted selectivity for JmjC KDMs recognising the same H3K9 methylation (for representative structure, see **67**, Fig. 8).

In another study, *N*-oxalyl-d-cysteine (DNOC) and a set of histone H3 peptides systematically substituted with cysteine residues were screened by techniques including non-denaturing mass spectrometry for binding to KDM4A and other KDM4 subfamily enzymes. Following identification of mixed disulfides that bound (for a view of a crystal structure on one cross-linked peptide–DNOC conjugate in the active site of KDM4A, see Fig. 10), stable analogues were synthesised (via radical ene reaction) resulting in an inhibitor selective for the KDM4 subfamily over KDM3A, KDM6B and KDM2A (**68**, Fig. 8) [141]. Moreover, by cross-linking to an H3K36 sequence (which binds to KDM4A–C but not D/E, **69**, Fig. 8), it was possible to demonstrate intra-subfamily selectivity (i.e. selectivity for KDM4A–C over KDM4D/E). Overall, these results provide proof of principle that selective inhibition of not only JmjC subfamilies, but also isoform subgroups, is achievable by utilising binding to both 2OG and substrate binding sites.

The development of small molecules that mimic histone substrate binding for the JmjC KDMs is at an early stage. The G9a and G9a-like H3K9 methyltransferase inhibitors BIX-01294 (**70**, Fig. 8) and E67 (**71**, Fig. 8) were shown to inhibit the H3K9me2 and H3K27me2 demethylase KDM7A with IC_50_ values in the low-micromolar range [142]. The E67-2 analogue (**72**, Fig. 8) retained potency against KDM7A with > 30-fold selectivity over G9a-like methyltransferases. All three compounds demonstrated selectivity for KDM7A over KDM5C. Both E67 and E67-2 displayed reduced cytotoxicity in mouse and human primary fibroblasts compared with BIX-01294. It is proposed that these compounds act as H3 substrate analogues as supported by crystallographic analyses of E67 complexed with KDM7A (Fig. 11). These studies imply further work on substrate-competing inhibitors of the JmjC KDMs will be worthwhile.

### 3.10 Inhibition by flavonoids, catechols and other compounds

Flavonoids and catechols are natural products that have been demonstrated to inhibit a number of 2OG oxygenases, including the JmjC KDMs (and many other enzymes) [109], [143], [144], [145]. A high-throughput screen of the LOPAC bioactive library against KDM4E identified many flavonoids (e.g. myricetin **73**, baicalein **74** and epigallocatechin gallate **75**, Fig. 9) and catechols (e.g. dopamine **76** and its derivatives, and haematoxylin **77**, Fig. 9) as low-micromolar inhibitors [146]. In another study, a catechol series (caffeic acid (**78**, Fig. 9) and derivatives) from a diversity-optimised natural product library was found to inhibit KDM4C and KDM6A but not PHF8 [147]. Caffeic acid **78** was a hit from a KDM6B HTS [148]. While the metal-chelating properties of flavonoids and catechols is, in part, thought to contribute to KDM inhibition, direct competition with 2OG is not observed, and it is possible that multiple factors contribute to their inhibitory effect. Although these compounds are unlikely to be useful from a pharmaceutical perspective, these results do raise the possibility that natural products obtained from the diet may affect chromatin modification status.

Recently, the natural product Tripartin (**79**, Fig. 9), which is a dichlorinated indanone isolated from the culture broth of the *Streptomyces* sp. associated with a larva of a dung beetle, has been reported as a JmjC KDM inhibitor; cell-based evidence for KDM4 inhibition was presented, though selectivity studies on isolated enzymes have not yet been reported [149].

The majority of JmjC KDM inhibitors identified to date incorporate carboxylic acids/carboxylic acid analogues, leading to use of pro-drug ester forms for sufficient cellular activity. Interestingly, a series of pyrido[1,2-a]indoles are reported to inhibit KDM4C in the sub-micromolar range as the acid, ester, and primary amide forms; the mode of action of these compounds is not yet established (Fig. 9) [150], [151].

## 4 ‘Pan’ KDM1 and JmjC KDM inhibitors

**Fig. 13:**
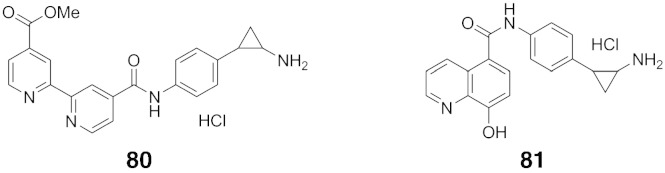
Structures of 'pan-KDM' inhibitors. Compounds **80** and **81** (as racemates) combine KDM1 inhibitor tranylcypromine **2** with the JmjC KDM inhibitors 4-carboxy-4′-carboxymethoxy-2,2′-bipyridine **51** and IOX1 **54** respectively. These dual inhibitors induce cell growth inhibition and apoptosis in prostate and colon cancer cell lines, but not in noncancer mesenchymal progenitor cells.

‘Pan’-histone demethylase inhibitors **80** and **81** (Fig. 13) targeting both KDM1 and JmjC KDMs were synthesised in an effort to address the observation that both KDM1 and KDM4 are coexpressed and colocalise with the androgen receptor in prostate cancer [152]. These inhibitors were created by covalently linking the known KDM1 inhibitor tranylcypromine (**2**, Fig. 2) with the JmjC KDM inhibitor templates 4-carboxy-2,2′-bipyridine (**51**, Fig. 4), or IOX1 (**54**, Fig. 4) [153]. Both **80** and **81** increased H3K4 and H3K9 methylation levels in cells and led to growth arrest and apoptosis in LNCaP prostate and HCT116 colon cancer cell lines; this activity was not observed with single **2**, **51** or **54**, or a combination of **2** and **51**. Further, the inhibitors were observed to cause little or no apoptosis in non-cancerous mesenchymal progenitor (MePR) cells. Thus, although there is undoubtedly considerable scope for optimisation, inhibitors targeting sets of both KDM1 and JmjC KDMs, may have potential for cancer-selective applications.

## 5 Future directions

In this review we hope to have summarised the pioneering progress on the inhibition of the human KDMs and given some sense of the excitement in the field from both basic science and therapeutic perspectives. Over the past decade or so our perceptions of the roles and extent of protein and nucleic acid methylation have changed considerably. A major change has come with the realisation that the methylation of protein, DNA and RNA molecules can be directly and frequently reversed by methyl group oxidation catalysed by enzymes from the amine oxidase and 2OG oxygenase superfamilies. A third mechanism of direct demethylation, i.e. by thiolate-mediated nucleophilic displacement, is involved in nucleic acid repair in both prokaryotes and eukaryotes, but has not been shown to be involved in transcriptional regulation [154].

Inhibition of histone demethylases and methyltransferases has substantial potential for the regulation of gene expression by treatment with small molecules. As outlined in the preceding sections, studies on the selective inhibition of the catalytic domain of both the human KDM1/LSD and the JmjC families of KDMs are progressing rapidly. Although these studies are at a relatively early stage, the signs are that with sufficient medicinal chemistry efforts, it will be possible to make highly potent and selective inhibitors of the catalytic domains of both families of human KDMs. To date most KDM1 and JmjC KDM inhibition efforts have been focused on the extension of known types of inhibitors for other family members to the KDMs, i.e. mechanism-based inhibition of the KDM1s and active site iron chelators for the JmjC KDMs. It is likely that the extension of those methods, including by competing with histone substrate binding interactions, will lead to highly selective inhibitors of the catalytic domains. As time progresses it is likely that new types of inhibitors will emerge — such as those involving substrate competition and allosteric interactions. Additionally, although more challenging, the identification of small molecules that promote KDM activity is of potential therapeutic interest and this might be the focus of some future academic efforts.

There is already strong evidence that the biological roles of the KDMs cannot be simply explained as ‘erasure’ of methylation. All human KDMs are predicted to contain non-catalytic ‘binding’ domains, and their interaction with nucleosomes/chromatin perhaps might be best viewed as protein–protein/nucleic acid interactions in which the covalent methylation/demethylation processes are a component. Perturbation of the interaction of the KDM binding domains offers potential for therapeutic manipulation. However, such an approach requires the issue of how best to assay KDM activity in cells.

To date, cell-based assays have mostly focused on antibody-based methods for measuring methylation status, sometimes coupled to ‘reporter gene’ assays, though not always under physiologically relevant conditions. Data relating to the methylation status associated with particular DNA sequences can also be acquired via chromatin immunoprecipitation studies. As with studies on other chromatin-modifying enzymes, in few cases are the effects of KDM inhibition directly connected with clear phenotypes at the physiological level. Mutations to the genes encoding for some KDMs are linked to pathophysiological phenotypes — and one interesting avenue will be to attempt to replicate these phenotypes using selective small-molecule inhibitors. Accumulating evidence, however, indicates that multiple post-translational modifications contribute to the emergence of phenotypes including pathophysiological ones.

Thus, small-molecule ‘epigenetic therapies’ may be required to inhibit multiple targets – at least for optimal therapeutic benefit – perhaps aimed at modulating sets of post-oligomerisation modifications associated with specific regions of chromatin. Achieving this in a rational manner will require substantially more knowledge of the chemical details of genetic processes than we presently possess and acquiring such detailed knowledge is likely to take some time — in the meantime it will be interesting to study the effects of combinations of inhibitors targeting different regulatory components of the (epi)genetic machinery.
